# Atomistic Insights into Ultrafast SiGe Nanoprocessing

**DOI:** 10.1021/acs.jpcc.3c05999

**Published:** 2023-09-27

**Authors:** Gaetano Calogero, Domenica Raciti, Damiano Ricciarelli, Pablo Acosta-Alba, Fuccio Cristiano, Richard Daubriac, Remi Demoulin, Ioannis Deretzis, Giuseppe Fisicaro, Jean-Michel Hartmann, Sébastien Kerdilès, Antonino La Magna

**Affiliations:** † CNR IMM, Z.I. VIII Strada 5, 95121 Catania, Italy; ‡ STMicroelectronics, Stradale Primosole 50, 95121 Catania, Italy; § Université Grenoble Alpes, CEA, LETI, 38000 Grenoble, France; ∥ LAAS-CNRS, Université de Toulouse, 31400 Toulouse, France; ⊥ Univ Rouen Normandie, INSA Rouen Normandie, CNRS, Normandie Univ, GPM UMR 6634, F-76000 Rouen, France

## Abstract

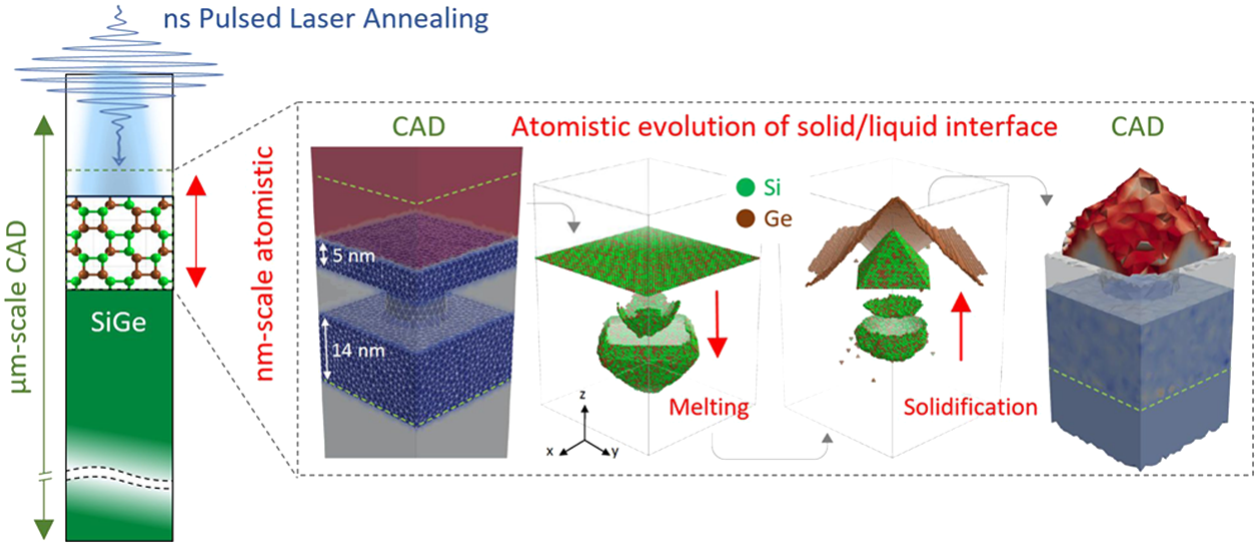

Controlling ultrafast material transformations with atomic precision is essential for future nanotechnology. Pulsed laser annealing (LA), inducing extremely rapid and localized phase transitions, is a powerful way to achieve this but requires careful optimization together with the appropriate system design. We present a multiscale LA computational framework that can simulate atom-by-atom the highly out-of-equilibrium kinetics of a material as it interacts with the laser, including effects of structural disorder. By seamlessly coupling a macroscale continuum solver to a nanoscale superlattice kinetic Monte Carlo code, this method overcomes the limits of state-of-the-art continuum-based tools. We exploit it to investigate nontrivial changes in composition, morphology, and quality of laser-annealed SiGe alloys. Validations against experiments and phase-field simulations as well as advanced applications to strained, defected, nanostructured, and confined SiGe are presented, highlighting the importance of a multiscale atomistic-continuum approach. Current applicability and potential generalization routes are finally discussed.

## Introduction

As materials science roadmaps relentlessly pursue the digital, sustainability, and quantum paradigms, understanding and harnessing ultrafast transformations at the atomic scale are becoming increasingly crucial for the atom-by-atom control of nanosystems and their integration as building blocks into meso- and macroscale systems.^1−5^ Laser annealing (LA) using excimer pulses is an excellent and long-standing way of inducing and investigating such transformations, as it enables localized energy absorption, heating, and melting over nanometer-sized subportions of the material in extremely short time (from tens to hundreds of nanoseconds).^5,6^ It is nowadays exploited in several technologies, mostly due to its ultralow thermal budget and its numerous control knobs (light wavelength and polarization, pulse duration, fluence, repetition rate, beam extension), which can be flexibly tuned to target specific functionalities, while handling the evergrowing complexity of nanosystems.

In the context of group IV elemental and compound semiconductor processing, pulsed-LA applications are ubiquitous.^5,7,8^ These include the fabrication of poly-Si thin-film transistors,^9−11^ ultrashallow device junctions,^10,12−14^ efficient contacts by silicidation,^15^ explosive crystallization,^16−18^ strain, defect,^19,20^ and dopant engineering.^21−25^ Localized heating minimizes the risk of damaging sequentially integrated components of monolithic three-dimensional (3D) devices.^26−30^ In optoelectronics, pulsed-LA is a key process for fabricating poly-Si displays,^31−34^ thin metal-oxides,^7^ pure-carbon electrodes for touch screens or solar cells,^35^ and hyper-doped semiconductors for near-infrared photodetectors.^36^ It also allows strain, composition and morphology engineering of fiber-based photonic devices,^8^ and fabrication of heavily doped superconducting silicon for monolithic quantum device integration.^23,37−39^ Despite all of these applications, understanding the ultrafast nonequilibrium kinetics of the liquid/solid interface in early stages of the process and correlating it to the postirradiation morphology and properties is challenging. This is because any experimental characterization,^5^ no matter how accurate, can only access the final state of the system. Observations would indeed require in situ, atomically resolved, and real-time capabilities well beyond those of modern electron microscopy^4^ or atom-probe facilities.^40^ For this reason, computer simulations are indispensable for both fundamental studies and the technological exploitation of LA.

LA simulations are usually deployed by self-consistently solving the electromagnetic interaction and heat diffusion problem in the irradiated system using a continuum description of its phase changes.^41−45^ LA process parameters can be explored and fine-tuned in micrometer-sized geometries with the aid of computational libraries that use finite-element methods (FEMs) to solve the underlying coupled partial differential equations. However, continuum models cannot capture local nanoscale changes in the annealed materials with atomistic resolution. The latter may be a critical factor, especially for compound materials with complex 3D geometries or phase diagrams, crystal-orientation-dependent kinetics, and defects, like stacking faults, which can affect regrowth. Polymorphic solidification may also introduce structural disorder in the form of intermixed stacking motifs (e.g., cubic, hexagonal).^46−48^ These phenomena can significantly alter the post-LA morphology and composition, with important consequences for device quality and performance. To ensure the appropriate process design and optimization, a simulation tool should be able to model the complex interplay between laser–matter interactions, the molten phase nonequilibrium kinetics, and all possible atomic-scale structural transformations while requiring the least amount of computational resources.

In this work, we present a multiscale computational methodology enabling simulations of LA processes with atomic resolution. It is based on the local self-consistent coupling of a state-of-the-art micrometer-scale FEM code with a kinetic Monte Carlo on superlattice (KMCsL) code able to simultaneously model atoms in the cubic and hexagonal crystal phases. Such a multiscale approach enables atomistic modeling of extended defects, shape changes, composition, and crystal phase adjustments affecting the laser-annealed material up to hundreds of nanometers below the surface while exchanging information between FEM and KMCsL at a nanosecond pace. In this way, it not only overcomes the limits of purely continuum-based tools but also overcomes those of other hybrid FEM-KMC approaches, which either lack self-consistent information exchange between the two frameworks^49^ or are limited to defect-free LA simulations of silicon without any superlattice formulation.^50^ In particular, we demonstrate the method by focusing on ultraviolet nanosecond-pulsed-LA processes of SiGe, an alloy with composition-dependent electronic and optical properties^51−53^ increasingly relevant to future nanoelectronic,^3,28,54−59^ thermoelectronic,^60^ optoelectronic,^8,61,62^ and quantum technologies.^2,63−65^ The multiscale methodology provides unique atomistic insights into the complex and ultrafast morphological, compositional, and structural transformations of SiGe during laser irradiation,^19,20,66,67^ giving invaluable support to process engineers aiming at the exploitation of this material’s full potential.

## Results and Discussion

### Multiscale FEM-KMCsL Coupling

Our LA simulations are based on a multiscale coupling between FEM and KMCsL solvers, which exchange information synchronously in a self-consistent loop at time steps Δ*t* < 1 ns throughout the simulation. Besides providing atomistic insights, this approach ensures higher accuracy compared to pure phase-field or enthalpy formalisms,^5^ as the latent heat exchanged at every Δ*t* is computed by direct integration of the volume subjected to a phase transition during each KMCsL step. The multiscale procedure is hereby described. After the appropriate 3D mesh was set up for a system with desired size and composition, the FEM calculation begins. The laser-induced heat source and temperature field *T*(*t*, **r**) within the irradiated material are self-consistently calculated by solving Maxwell’s and Fourier’s partial differential equations. As the system absorbs energy from the laser pulse, following its power density modulation, the surface temperature increases until local melting occurs. This initiates the feedback coupling with KMCsL, which models atom-by-atom the concerned system subregion. The following steps are then iterated every Δ*t* over the whole pulse duration:

- *T*(*t*, **r**) is interpolated into the dense KMCsL superlattice and defines melting/solidification event probabilities;
- KMCsL simulates the evolution of the solid/liquid (S/L) interface for a time Δ*t* with the established probability table, capturing atomic-scale structural adjustments, lattice faceting, vacancies, extended defects, polymorphic solidification, and species redistribution;
- the S/L volumes and the local species concentrations are updated in the mesh based on the KMCsL results and affect the *T*(*t*, **r**) calculation in the subsequent FEM cycle.

**Figure 1:**
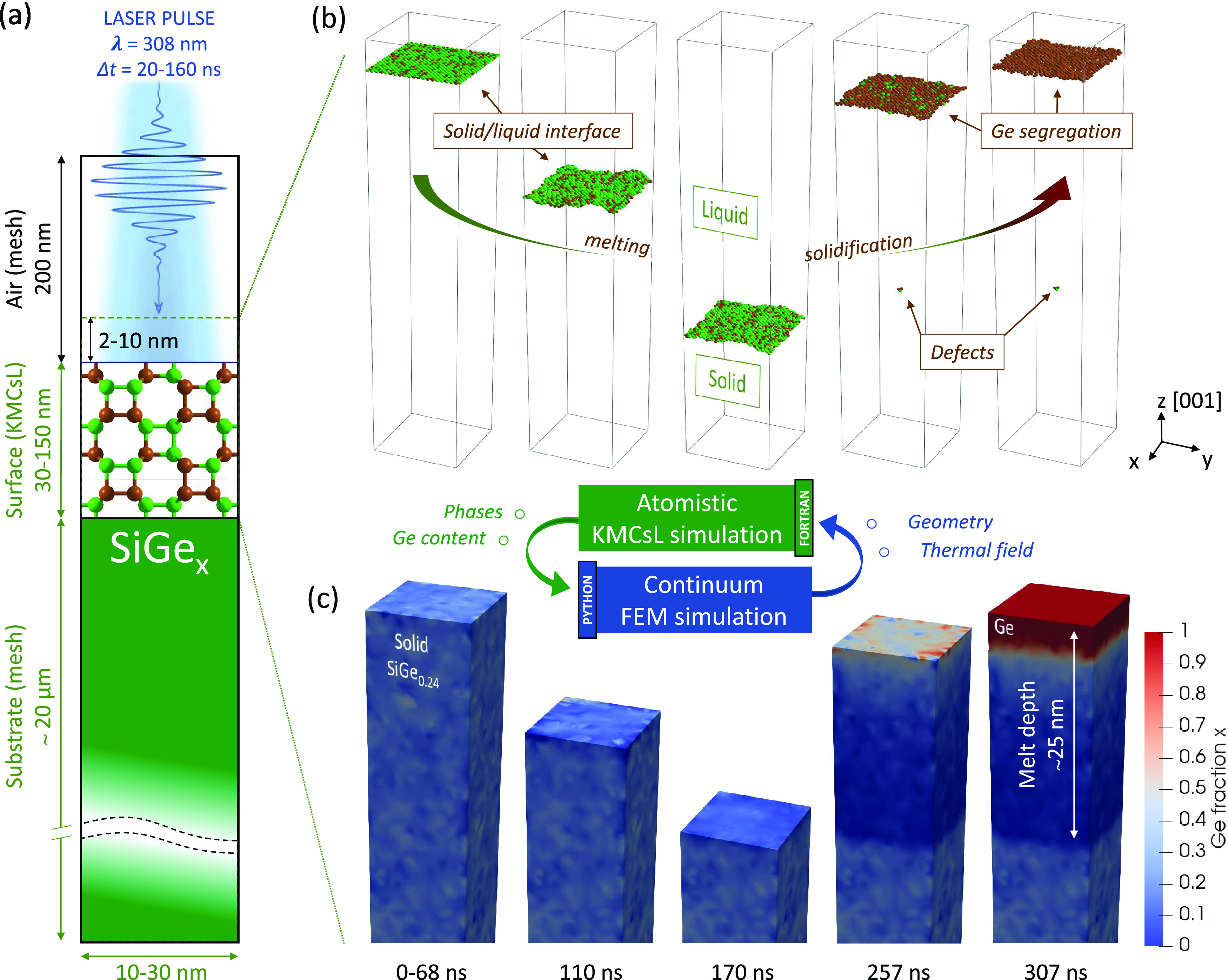
Schematics of the FEM-KMCsL multiscale approach applied to a SiGe (0 0 1) surface. (a) Sketch of the simulation framework. Typical simulation box dimensions are also indicated. (b) KMCsL model at various instants during melting and solidification. The KMCsL box initially includes 2–10 nm of air and the top 30–150 nm of the surface. Solid undercoordinated Si and Ge atoms (green and brown, respectively) identify the solid–gas interface in the first and last snapshots and the solid–liquid interface in the intermediate ones. (c) Ge content in solid SiGe in the FEM model, visualized at the same instants. Pure Si (*x* = 0) regions are shown in blue and pure Ge (*x* = 1) regions in red.

The above three steps are iterated until all previously melted atoms resolidify. Thereafter, the FEM-KMCsL communications stop, and the FEM model is left to cool. Figure 1 schematically illustrates a typical FEM-KMCsL simulation box (characteristic sizes used in this work are also indicated) for modeling pulsed-LA of a flat Si_0.76_Ge_0.24_ (0 0 1) surface. Figure 1b shows the solid atoms at the S/L interface in the KMCsL-modeled subregion at various instants of a simulation assuming a XeCl excimer (λ = 308 nm) 160 ns laser pulse, with a 0.75 J cm^–2^ energy density and a Δ*t* = 0.25 ns. After the initial heating stage up to *T*_M_(*x* = 0.24) ≈ 1573 K, the interface goes deep into the material (roughly 25 nm), keeping a roughness of a few nanometers. Then, it rapidly ascends as *T* decreases, solidifying a SiGe layer with a graded Ge content and a Ge-rich surface, due to nonequilibrium species partitioning in the alloy.^68^ The corresponding solid-phase regions in the 3D FEM mesh at the same instants are reported in Figure 1c, with colors highlighting Ge segregation.

More theoretical and technical details about the multiscale implementation are reported in the Supporting Information (see Supporting Information S1 and S2, Figure S1, and Movie S1).

### Calibration of KMCsL for the SiGe S/L Interface

**Figure 2:**
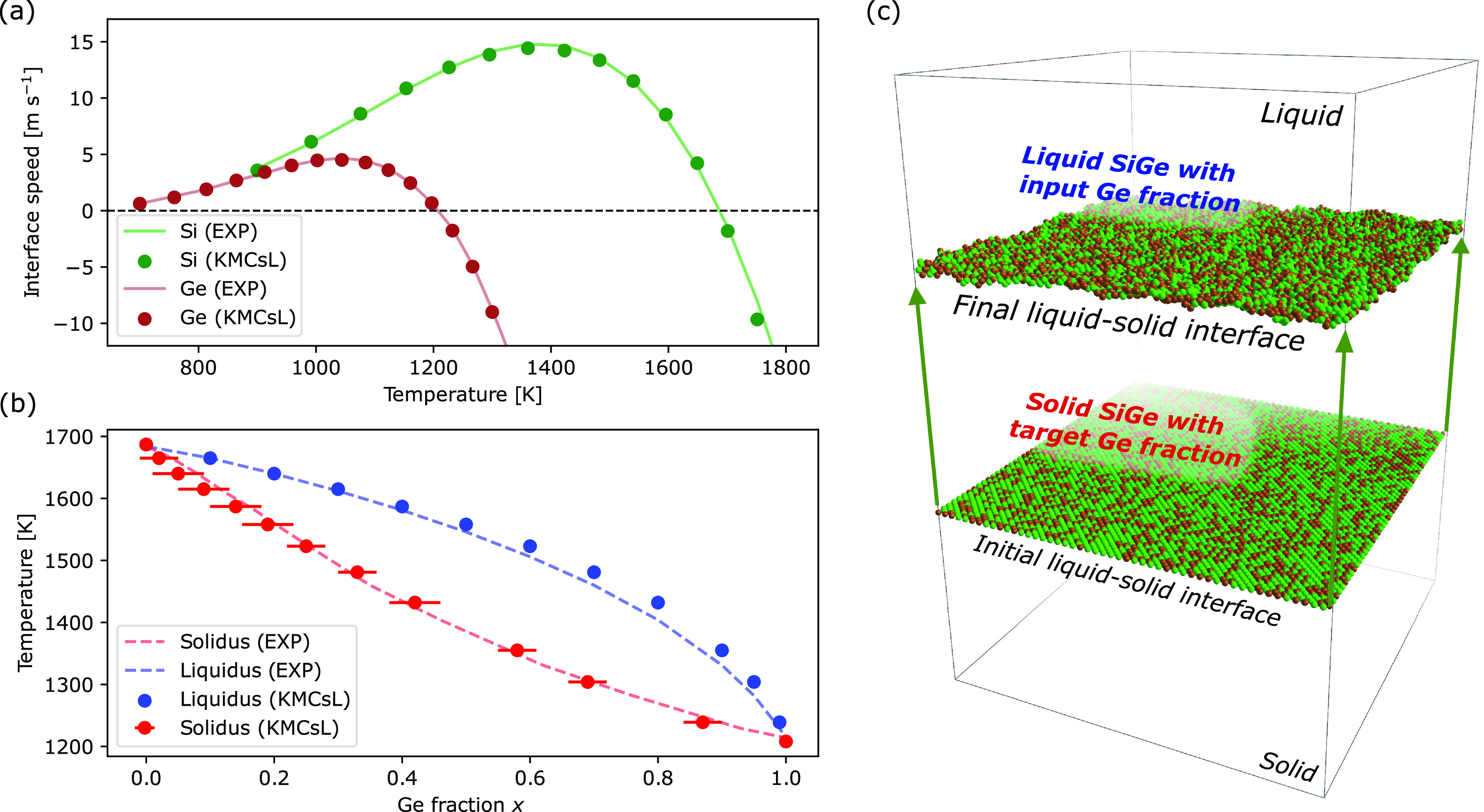
KMCsL calibration for SiGe solid–liquid-phase transitions. (a) Calibrated results for the solid–liquid interface velocity in pure Si and pure Ge as a function of temperature (markers) in comparison with the respective Fulcher–Vogel profile (lines). (b) Calibrated results for the SiGe phase diagram (markers), along with the expected phase diagram (dashed lines). The horizontal bars reflect spatial compositional variations in the solidified layer. (c) Schematics of the strategy followed for phase diagram calibration. The superimposed snapshots show initial and final states of a calibrated simulation for *T* ≈ 1410 and *x*_L_ ≈ 0.8, resulting in *x*_S_ ≈ 0.43. Solid undercoordinated Si and Ge atoms (green and brown, respectively) identify the interface.

In the KMCsL model of a partially melted system, the evolution of the S/L interface is governed by the balance between solidification and melting events with *T*-dependent Arrhenius-like probabilities (see Supporting Note S1). To ensure reliable LA simulations, it is crucial to calibrate the KMCsL event probabilities so that they reproduce the correct S/L interface kinetics over a wide range of temperatures. In the case of SiGe alloys, this calibration is carried out in two steps. The first, following the strategy of ref (50), consists of reproducing the Fulcher–Vogel curves of pure Si and Ge systems,^16,69^ i.e., the S/L interface velocity as a function of *T*. We perform this by initializing solid Si and Ge surfaces surmounted by an infinite liquid reservoir and performing a sequence of KMCsL simulations for a wide range of *T* around *T*_M_, always assuming uniform *T* in the simulated box. The KMCsL parameters are then fine-tuned to yield the expected interface velocities, as shown in Figure 2a (calibrated values in Supporting Tables S1–S3). The second step, since no Fulcher–Vogel relation holds for SiGe alloys, consists of calibrating the KMCsL event rates involving mixed Si–Ge bonds on the SiGe phase diagram^51^ (dashed lines in Figure 2b), which describes the *T*-dependent composition (*x*_S_, *x*_L_) of solid and liquid phases at equilibrium, when the melting/solidification process occurs very slowly. This is achieved by setting up slightly undercooled KMCsL simulations around a given (*T*, *x*_L_) point in the phase diagram and tuning the parameters until the solidified SiGe layer roughly matches the expected *x*_*S*_ (more details in Supporting Note S3). For example, Figure 2c shows two snapshots of the S/L interface at the beginning and at the end of a well-calibrated simulation (*T* ≈ 1410, *x*_L_ ≈ 0.8). This predicts a solid layer with an average *x*_S_ ≈ 0.43, which is in line with the phase diagram and hence confirms the reliability of the calibration at the considered *T*. The simulated results spanning the entire phase diagram from Si-rich to Ge-rich situations are reported in Figure 2b.

### Validation of Multiscale LA Simulations for Relaxed/Strained SiGe

Here, we show the results of FEM-KMCsL simulations for relaxed and 30 nm thick strained SiGe (0 0 1) layers epitaxially grown on Si. To check the consistency of the method and validate it, we compare these results with those of one-dimensional (1D) nonatomistic simulations based on a state-of-the-art FEM-phase-field formulation (see Supporting Note S1), considering pulsed-LA processes with various laser energy densities and pulse durations (160 ns for relaxed, 146 ns for strained). The simulation settings (optical/thermal parameters, initial Ge profile, laser properties) in the 1D purely continuum and the 3D FEM-KMCsL multiscale frameworks are identical, except for the mesh dimensionality and the formalism describing phase transitions (phase field with a smooth S/L interface in one case, KMCsL with an atomically sharp S/L interface in the other).

**Figure 3:**
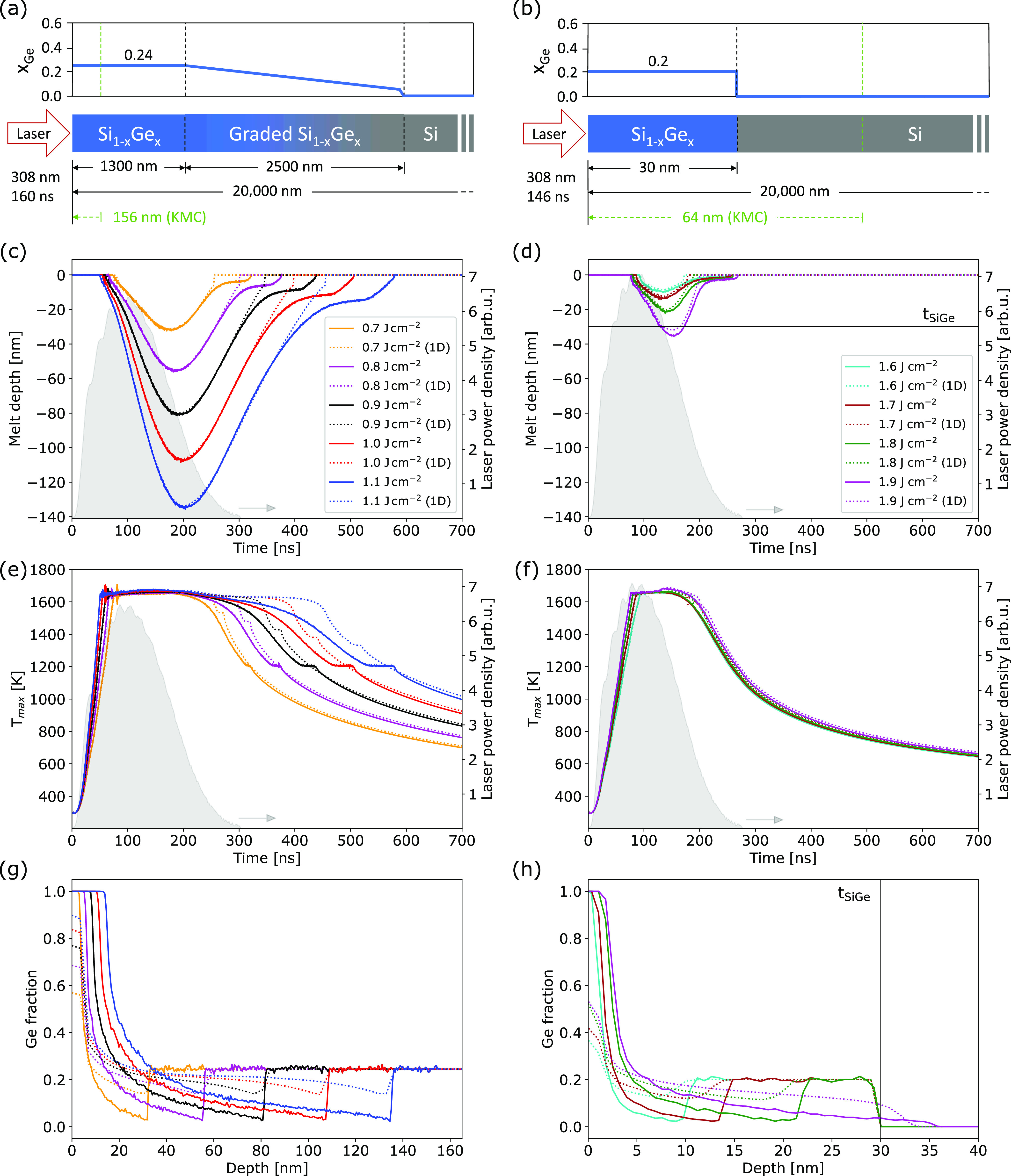
Comparison between atomistic FEM-KMCsL (solid lines) and nonatomistic phase-field simulations (dotted lines). (a, b) Pulsed-LA simulation setup for relaxed (left) and strained SiGe (right) with thickness *t*_SiGe_ = 30 nm, including system sizes and initial composition. (c, d) Time variation of melt depth, (e, f) maximum temperature in mesh, and (g, h) postanneal Ge profiles obtained for various laser energy densities. The laser pulse shape (filled gray areas) and power density (right axes) are also reported.

The initial Ge profiles and process conditions are listed in Figure 3a,b. Figure 3c,d shows that the two methodologies yield almost identical results concerning the general melt depth profile over time, with KMCsL yielding melting/solidification velocities and maximum melt depths in remarkably good agreement with phase-field simulations. Contrary to the latter, the FEM-KMCsL approach can track the interface evolution up until the last solidification event, and as a result, it can reproduce the expected slowdown of the solidification front due to the gradual Ge incorporation. Figure 3e,f shows the variation of the maximum temperature *T*_max_ in the mesh over the same time interval. Both models predict an overall similar trend, with the expected change in slope at the onset of melting and almost overlapped cooling tails after complete solidification. The observed *T*_max_ plateaus at the end of solidification are also related to Ge segregation, as those observed in Figure 3c,d. Their position and dependence on energy density differ between the two models, in accordance with the final profiles of Ge concentration over depth reported in Figure 3g,h. Like all other noticeable deviations in Figure 3, this can be attributed to intrinsic differences between the two models (more details in Supporting Note S4). Overall, these results confirm the internal consistency of the multiscale methodology for a wide range of process conditions and demonstrate that the phenomenon of Ge segregation is qualitatively captured in both relaxed and strained SiGe.

**Figure 4:**
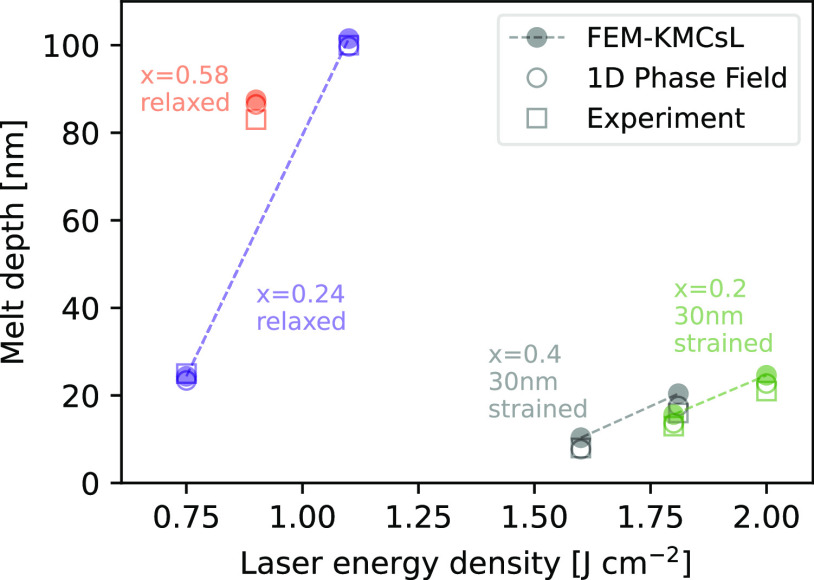
Comparison between maximum melt depths simulated with FEM-KMCsL (filled circles), 1D phase field (empty circles), and experimental measurements (squares) for various energy densities and Ge fractions. Relaxed (strained) samples are irradiated with a 308 nm, 160 ns (146 ns) pulse. Dashed lines guide the eye through processes on the same sample.

Further validation is provided by the results in Figure 4. These demonstrate that the maximum melt depths obtained with FEM-KMCsL simulations are also in good agreement with 1D phase-field simulations and experimental measurements (experimental details are reported in Supporting Note S1). In particular, here we consider processes with 160 ns pulses for relaxed Si_0.76_Ge_0.24_ and Si_0.42_Ge_0.58_ and with 146 ns pulses for strained 30 nm thick Si_0.8_Ge_0.2_ and Si_0.6_Ge_0.4_. The maximum melt depths and Ge fraction for the relaxed samples were extracted from energy-dispersive X-ray spectroscopy measurements performed after the irradiation. Data for strained samples is taken from refs (19,66). We note that the agreement could be improved by further tuning of the calibration parameters.^70^

### LA Simulations with Stacking Faults

**Figure 5:**
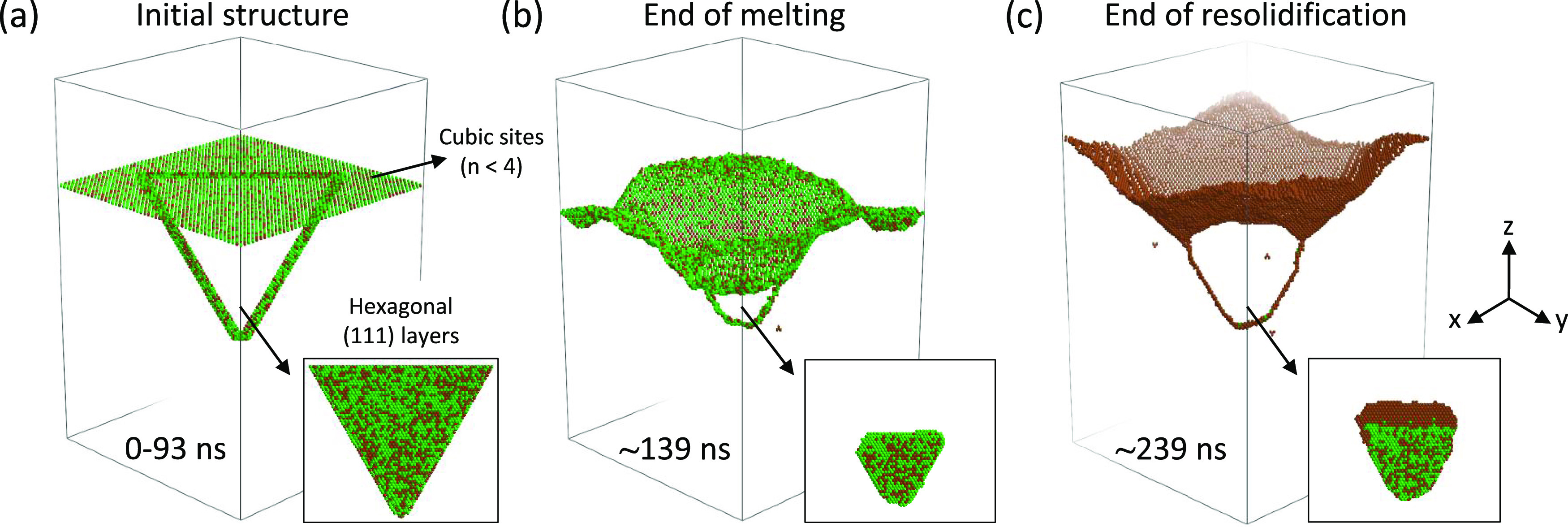
Multiscale LA simulations (308 nm, 146 ns, 1.3 J cm^–2^) for strained Si_0.6_Ge_0.4_ on Si with a pre-existing triple stacking fault. Undercoordinated cubic solid atoms in the KMCsL box are shown at three instants during the process: (a) before melting, (b) at the maximum melt depth, and (c) at the end of solidification. Hexagonally stacked atoms (regardless of coordination) at all instants are shown in the insets.

The KMCsL formulation allows the study of the impact of extended stacking defects on the final morphology of laser-annealed materials (see Supporting Note S1). As an example, in Figure 5, an LA process for strained 30 nm Si_0.6_Ge_0.4_ on Si is considered (146 ns, 1.3 J cm^–2^), where an ∼10 nm deep triple stacking fault exists in the sample prior to laser irradiation. This introduces three hexagonally stacked (1 1 1) atomic layers into the cubic SiGe structure. All cubic undercoordinated surface atoms in the KMCsL box before melting are shown in Figure 5a, along with the bulk ones enclosing the (1 1 1) layers. The S/L interface at the maximum melt depth and after full solidification is shown in Figure 5b,c (see also Supporting Movie S2). We find that part of the defect is melted along with 7–8 nm of SiGe, without significant impact on the melting kinetics, and that a strongly inhomogeneous solidification is triggered by the unmelted hexagonal sites. Liquid atoms in direct contact with them indeed solidify much slower than the others, favoring the {1 1 1} faceting of the S/L interface (see Figure 5a–c). Segregation is observed in both cubic and hexagonal crystal phases (see the insets in Figure 5). For higher energy densities, the defect is fully melted and a purely cubic phase planar solidification occurs as usual.

### LA Simulations with Nanostructured and Constrained Geometries

**Figure 6:**
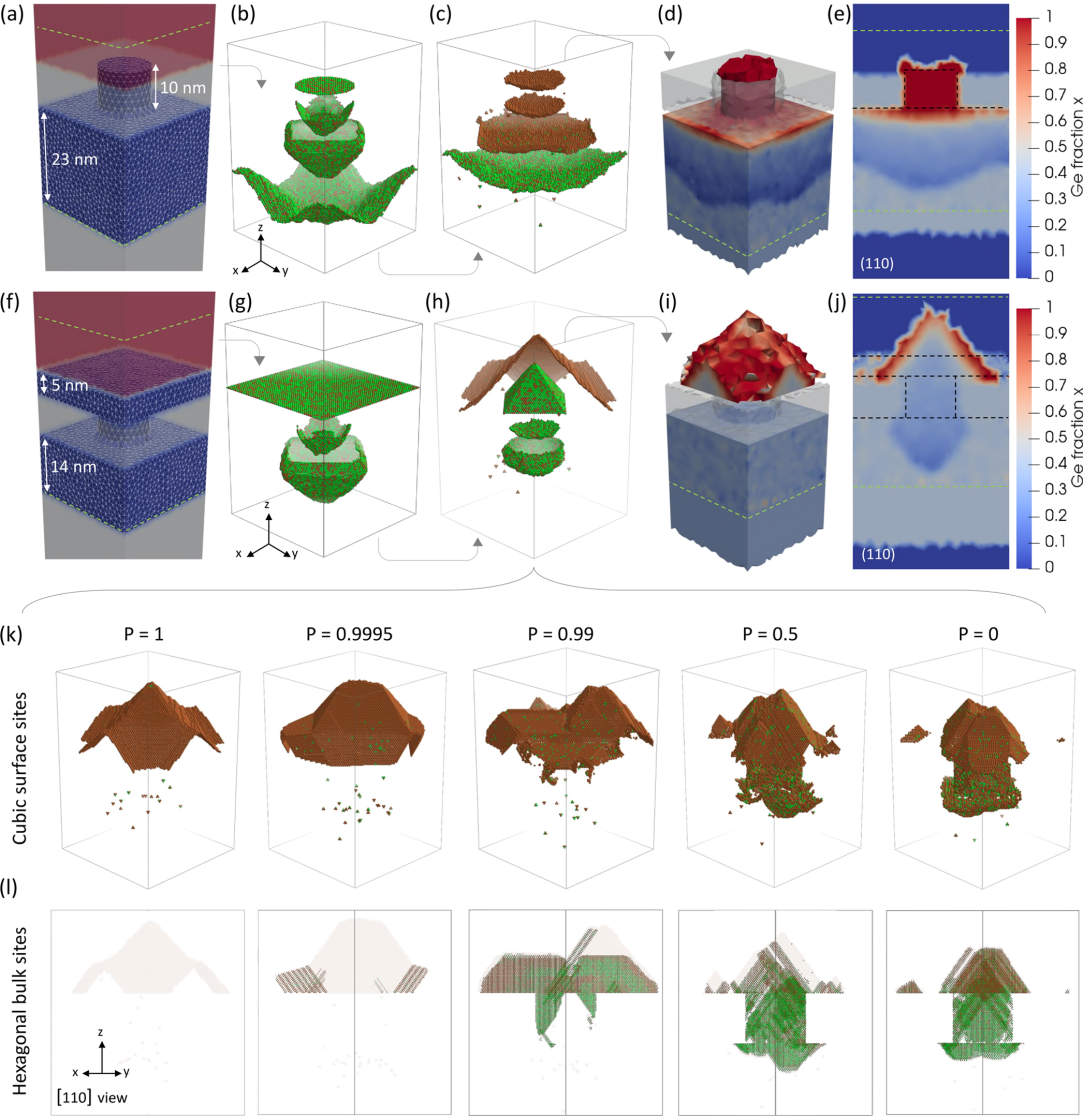
(a–e) Pulsed-LA simulation (308 nm, 22 ns, 0.95 J cm^–2^) of 30 nm strained Si_0.6_Ge_0.4_ with 9 nm large and 10 nm high Si_0.6_Ge_0.4_ NWs on top, embedded in nonmelting SiO_2_. (a) Input FEM periodic mesh. KMCsL-coupled SiGe regions are shown in blue, air in red, and SiO_2_ and non-KMCsL-coupled regions in gray. The KMCsL cell extension along *z* is indicated with dashed green lines. (b) Overlapped selected snapshots of the liquid–solid interface in the KMCsL box at various instants during melting and (c) solidification. Green (brown) spheres indicate Si (Ge) atoms. (d) 3D view and (e) (1 1 0) cut-plane of the final Ge distribution in the FEM mesh. Regions outside the KMCsL cell (below the green dashed line) appear uniformly colored because no KMCsL mapping occurs therein. The initial surface morphology is indicated by dashed black lines. (f–j) Simulation at a 1.2 J cm^–2^ energy density for the same system as above, including a 5 nm thick Si_0.6_Ge_0.4_ capping layer. (k) Cubic undercoordinated sites in the KMCsL box at the end of the simulations performed at the same conditions as (f–j) but with different polymorphic solidification probabilities *P*. (l) Hexagonally stacked sites (regardless of coordination) from the simulations in panel (k), viewed along the [1 1 0] direction. Cubic sites from panel (k) are redrawn in semitransparency.

The previous example reveals another important feature of KMCsL, i.e., the crystal-orientation-dependent kinetic evolution of the S/L interface. Such an atomistic feature is essential to model LA of SiGe systems with nanostructured and/or constrained geometries, which often involve reshaping and faceting of the solid–liquid interface throughout the process. An example is illustrated in Figure 6a–e. It considers an LA process (22 ns, 0.95 J cm^–2^) of a SiGe system similar to those used in vertical nanostructured channel arrays,^55^ namely, a 30 nm thick strained Si_0.6_Ge_0.4_ on Si with a 9 nm large and 10 nm high Si_0.6_Ge_0.4_ nanowire on top. The latter is embedded in SiO_2_, which does not melt during irradiation and therefore represents a geometrical constraint for the evolving S/L interface. An energy density of 0.95 J cm^–2^ is chosen to keep melting within the KMCsL box, which is 27 × 27 × 41 nm^3^ and includes ∼23 nm of the SiGe layer, the nanowire, the oxide, and ∼8 nm of air (see green dashed lines in Figure 6a). Figure 6b,c shows snapshots of the S/L interface at various instants during melting and solidification (see also Supporting Figure S3 and Movie S3). The circular nanowire tip exposed to the laser rapidly absorbs heat and melts all the way to the bottom of the oxide. In this process, the shape of the S/L interface is already {1 1 1}-faceted. After the complete melting of the nanowire, the nanodroplet reshapes into a half-octahedron below the oxide, which then coalesces with its periodic images, giving rise to a rough liquid layer, similar to what occurs in simulations of Si LA processes assuming inhomogeneous nucleation.^50^ Thereafter, the S/L interface flattens and moves toward the initial surface level. While constrained by the oxide, Ge segregation occurs, causing a total transformation of the initial SiGe nanowire to a pure Ge nanowire. The final Ge distribution in the FEM mesh is illustrated in Figure 6d,e as a (1 1 0) cut-plane. These figures show the rough shape of the interface at the maximum melt depth and highlight a slight tendency toward solidification of the nanowire shell before its core (also noticeable in the KMCsL snapshots).

By initializing the above simulation with an additional 5 nm thick Si_0.6_Ge_0.4_ capping layer (see Figure 6f), we trigger solidification on top of the nanowire/oxide region and give rise to more pronounced solid-phase reshaping effects. This time, we included ∼14 nm of SiGe, the nanowire, the oxide, the capping layer, and ∼11 nm of air within the KMCsL box and used 1.2 J cm^–2^ as energy density (enough to avoid coalescence of molten nuclei). The kinetic evolution in Figure 6g,h (see also Supporting Movie S4) and the final Ge distributions in Figure 6i,j reveal that the S/L interface initiates solidification with a nonplanar shape and assumes a highly symmetrical {1 1 1}-faceted pyramidal shape as it emerges above the oxide. This solid seed gradually expands and partially coalesces with its periodic replicas while concurrently segregating Ge.

### LA Simulations with Polymorphic Solidification

As a further demonstration of the potential of KMCsL for LA simulations, in Figure 6k,l, we report on the results of the previous simulation obtained while allowing for polymorphic cubic-hexagonal stacking transitions during solidification. Figure 6k depicts the cubic undercoordinated KMCsL sites at the end of LA simulations performed by varying the probability *P* of switching the stacking order (see Supporting Note S1). Figure 6l shows all hexagonally stacked atoms superimposed to the cubic ones (semitransparent), viewed along the [1 1 0] direction to highlight the presence of {1 1 1} atomic layers. We find significantly intermixed stacking motifs, even for very small probabilities. A high concentration of stacking faults (both single and triple) characterizes the oxide-embedded region, suggesting a clear correlation between confinement and stacking disorder. Noteworthily, the pyramidal shape of the final surface is quite robust against polymorphic disorder (see also Supporting Figure S3 and Movies S5 and S6).

## Conclusions

We have presented a new multiscale approach to model LA processes of group IV materials and alloys, including complex 3D shape modifications, faceting, species redistribution, stacking disorder, and extended defects. It is based on the self-consistent, parallel, and synchronous coupling of a continuum FEM-based solver for light–matter interaction and thermal diffusion with a KMCsL code. The latter simulates the kinetic evolution of the liquid–solid interface and lattice defects in a local region of the material with atomic resolution, enabling studies so far inaccessible to purely continuous simulation approaches. We point out that describing compound materials like SiGe and including the impact of off-lattice defects (like stacking faults and interstitial-like defects) during LA are the main improvements with respect to previously reported multiscale methods.^50^ In particular, we have described the theoretical background and computational implementation of the methodology in light of its application to the Si_1–*x*_Ge_*x*_ alloy, which represents one of the most promising candidates for 3D sequentially integrated devices,^28,56−58^ spin-qubits,^65^ gate-all-around transistors,^54,55,59^ or even direct-band-gap light emitters.^61^ The method was validated by comparing simulations for both relaxed and strained SiGe with the 1D phase-field results and experiments. It quantitatively reproduces the same melt depth profiles and qualitatively captures the laser-induced Ge redistribution. KMCsL has the advantage of avoiding the typical numerical instabilities of approaches based on the phase field, especially at the onset and the end of melting. The code was applied to simulate pulsed-LA processes of blanket and nanostructured SiGe systems including effects of extended defects and geometrical constraints. The possibility of studying the impact of extended defects and polymorphic solidification was demonstrated, and a clear correlation between bulk structural disorder and postirradiation surface morphology was observed.

Importantly, the methodology is implemented into an open-source versatile tool that offers several opportunities in terms of potential generalizations. The unique KMCsL superlattice framework, enabling the coexistence of multiple crystal arrangements in the same simulation box, is readily applicable to other elemental or compound group IV semiconductors with sp^3^ bond symmetry, e.g., Si, Ge, or SiC.^50,71^ By tailoring the crystal symmetries of the KMCsL lattice, it could be generalized to other binary alloys (e.g., GeSn),^72^ compound semiconductors (e.g., GaAs, AlGaAs), or polymorphic metal/semiconductor systems (e.g., NiSi, PtSi). Future KMCsL developments may broaden the kinetic landscape by including liquid-phase diffusion events and strain relaxation events. With properly calibrated FEM optical and thermal parameters, lasers with different wavelengths could be studied. Continuous-wave and scanning LA processes could also be investigated by adjusting the input profile of the laser power density.

Framing the FEM-KMCsL strategy into a broader multiscale perspective, one may envision advanced coupling with other *ab initio*, molecular dynamics, or transport simulation tools, e.g., to account for strain relaxation and interactions between extended defects^73^ or investigate the impact of ultrafast processing on device components.^74^ A similar multiscale approach could be used to study processes where other physical variables govern the atomic kinetics (e.g., strain, charge, polarization, magnetization) or where phase transitions are triggered by different ultrafast external stimuli (e.g., electric, magnetic, or strain perturbations).^75^ This could provide interesting insights into various research areas, from silicidation^15^ to multiferroics^76^ or phase-change resistive-switching materials for neuromorphic computing and high-speed photonic-based devices.^77,78^

In conclusion, we hope that our open-source simulation framework will serve as a useful support and source of inspiration for computationally assisted advanced characterization based on novel in situ and operando upgrades of electron microscopes.

## Data Availability

The minimal input data needed to replicate the findings reported in the article is available on Github and can be accessed via this link: https://github.com/MulSKIPS/MulSKIPS/tree/main/examples.

## Supporting Information Available

Additional details on the theoretical frameworks and experimental details (Note S1); the technical implementation of the FEM-KMCsL method in MulSKIPS (Note S2); details on the KMCsL calibration (Note S3); the differences between the 1D phase-field and the FEM-KMCsL approaches (Note S4); the corrections to the liquid SiGe dielectric constant (Note S5); and more details on the nanowire-based LA simulations (Note S6) (PDF)
Flat-Si_0.76_Ge_0.24_-160 ns-0.75 J cm^–2^ (Movie S1) (MP4)
Stacking fault-Si_0.6_Ge_0.4_-146 ns-1.3 J cm^–2^ (Movie S2) (MP4)
Nanowire-Si_0.6_Ge_0.4_-22 ns-0.95 J cm^–2^ (Movie S3) (MP4)
Capped nanowire-Si_0.6_Ge_0.4_-22 ns-1.2 J cm^–2^ (Movie S4) (MP4)
Polymorphic-capped nanowire-Si_0.6_Ge_0.4_-22 ns-1.2 J cm^–2^ (Movie S5) (MP4)
HexPolymorphic-capped nanowire-Si_0.6_Ge_0.4_-22 ns-1.2 J cm^–2^ (Movie S6) (MP4)

## Notes

The methodology presented in this work is implemented in the open-source tool MulSKIPS available on Github and can be accessed via this link: https://github.com/MulSKIPS.
