# Supplementary material for: Atomistic Insights into Ultrafast SiGe Nanoprocessing
Source: J Phys Chem C Nanomater Interfaces. 2023 Sep 27;127(39):19867–77. doi: 10.1021/acs.jpcc.3c05999 (PMC10561275; doi:10.1021/acs.jpcc.3c05999)
Supplement: Supplementary file 1 — jp3c05999_si_001.pdf [file jp3c05999_si_001.pdf]

Atomistic Insights Into Ultrafast SiGe Nanoprocessing
# - Supplementary Information -

Gaetano Calogero,[1,*] Domenica Raciti,[2] Damiano Ricciarelli,[1] Pablo Acosta-Alba,[3] Fuccio Cristiano,[4] Richard Daubriac,[4] Remi Demoulin,[5] Ioannis Deretzis,[1] Giuseppe Fisicaro,[1] Jean-Michel Hartmann,[3] Sébastien Kerdilès,[3] and Antonino La Magna[1,*]

[1] *CNR IMM, Z.I. VIII Strada 5, 95121 Catania, Italy*

[2] *STMicroelectronics, Stradale Primosole 50, 95121 Catania, Italy*

[3] *Université Grenoble Alpes, CEA, LETI, 38000 Grenoble, France*

[4] *LAAS-CNRS, Université de Toulouse, 31400 Toulouse, France*

[5] *Université Rouen Normandie, 76801 Saint Etienne Du Rouvray, France*

(E-mail: gaetano.calogero@imm.cnr.it; antonino.lamagna@imm.cnr.it)

NOTE S1. METHODS

Multiscale implementation

The multiscale simulation tool developed in this work is distributed as part of the open-source MulSKIPS simulation package [1, 2]. This includes a core KMCsL code built on a peculiar super-lattice framework which enables simultaneous modelling of cubic and hexagonal crystal phases in the same simulation cell. Such a functionality is critical for LA simulations of multi-element systems including non-ideal stacking and polymorphism. With the appropriate particle/event definition and calibration, it can also simulate epitaxial growth by physical or chemical vapour deposition [3]. The KMCsL model, coded in Fortran, is internally coupled to a FEM-based solver coded in Python with the Dolfin interface of the FEniCS computing platform [4] (the same solver is used for the benchmark 1D phase-field simulations). The PyMulSKIPS Python library, distributed with MulSKIPS , manages all simulation workflows and includes an I/O interface coupling the KMCsL simulator to the FEM solver and external Technology Computer-Aided Design (TCAD) tools. In particular, *ad-hoc* Application Programming Interfaces are implemented to manage the multi-process shared-memory execution of simulations via F2Py sockets [5, 6]. This ensures a real-time communication of all relevant geometrical and physical information between the different simulation frameworks. By allowing a single KMCsL process to run over the entire simulation, it also enables a continuous tracking of species position and bonding configurations, which is crucial for simulating the evolution of extended defects or polymorphic domains across consecutive FEM-

KMCsL cycles (more details in Supplementary Note S2).

KMCsL model

The KMCsL model is defined on a dense cubic super-lattice able to accommodate both cubic and hexagonal diamond lattices as sub-lattices. The super-lattice constant is $a_{\text{KMCsL}} \equiv a/12 \equiv l/\sqrt{27}$, with a the diamond lattice constant and l its nearest neighbour distance (0.543 nm and 0.235 nm for Si, respectively). This definition makes it readily applicable to any elemental, compound and alloy material with sp^3 (tetrahedral) bond symmetry, such as Si, Ge or SiGe, including non-ideal stacking configurations. Each super-lattice site is marked as either solid or liquid site and can have coordination $n \leq 4$ (for $n = 4$ they are marked as bulk). In case of $Si_{1\text{-x}}Ge_{\text{x}}$, the two atomic species are randomly allocated in the lattice of the input structure, reflecting the user-defined Ge fraction x. To reduce calibration parameters and memory consumption, thus improving scalability, a real atomic occupancy is strictly considered only in the solid phase (the accuracy of this approach was already demonstrated for Si LA simulations [7]). In case of SiGe alloys, while the solid-phase Ge fraction can be described as a local time-dependent variable, $x_S \equiv x_S(\mathbf{r}, t)$, the liquid-phase Ge fraction is averaged over the liquid volume at each ns-long KMCsL cycle $x_L \equiv x_L(t)$ (more details in Supplementary Note S4). In a partially-melted system, the kinetic evolution of the liquid-solid interface is governed by the balance between solidification and melting events. These are stochastically selected by a continuous time algorithm [7] and only involve under-coordinated ($n < 4$) super-lattice sites. We note that no kinetics occurs in the bulk, no matter if solid or liquid. Diffusion events are not explicitly defined in the KMCsL framework, but they can be effectively reproduced by close melting/solidification events nearby the interface. The solidification and melting event rates are thermally activated and therefore obey Arrhenius-like expressions, with prefactors and exponents depending on temperature T and bond coordination n [7]. In the case of SiGe alloys, they also explicitly depend on the fraction X^i of individual species in the liquid phase, with $X^i = x_L$ for Ge and $X^i = 1 - x_L$ for Si. In particular, the solidification (melting) event rate ν^i_{LS} (ν^i_{SL}) for species $i =$ Si,Ge on a site with $n = n_{\text{Si}} + n_{\text{Ge}}$ solid neighbours is defined as:

$$\nu^i_{\text{LS}} = f^i(T) \cdot X^i \cdot \nu^i_0 \cdot \exp\left(-\frac{2E^i_{\text{LS}}(n)}{k_B T^i_M}\right) \tag{S1}$$

$$\nu^i_{\text{SL}} = \nu^i_0 \cdot \exp\left(-\frac{nE^i_{\text{SL}}(n_{\text{Si}}, n_{\text{Ge}})}{k_B T}\right) \tag{S2}$$

where ν^i_0 is a species-dependent constant prefactor, T^i_M is the melting temperature of species i and k_B is the Boltzmann constant. The solidification rate increases with X^i and a damping term

$f^i(T) = 1/2[1 + \mathrm{erf}((T - T_0^i)/A^i)]$, with T_0^i and A^i adjustable parameters, effectively models the reduction in solidification velocity under strong undercooling, as predicted by the Fulcher-Vogel expression [8, 9]. $E_{\mathrm{LS}}^i(n)$ is the n-dependent solidification energy barrier for species i, whereas $E_{\mathrm{SL}}^i(n_{\mathrm{Si}}, n_{\mathrm{Ge}})$ is the melting energy barrier, which is equivalent to a binding energy and hence depends on the number and identity of nearest neighbours. We note that the event rates $\nu_{\mathrm{LS}}^i \equiv \nu_{\mathrm{LS}}^i(t, \mathbf{r})$ and $\nu_{\mathrm{SL}}^i \equiv \nu_{\mathrm{SL}}^i(t, \mathbf{r})$ vary with time t and lattice position $\mathbf{r}$ during the LA simulations. This stems from the fact that $T \equiv T(t, \mathbf{r})$ as well as $X^i \equiv X^i(t)$ are evaluated at every FEM-KMCsL cycle. All parameters in Equation (S1) are determined by calibrating pure Si and Ge systems against their Fulcher-Vogel curves (see Figure 2a in the main text). The melting energy barriers, $E_{\mathrm{SL}}^i(n_{\mathrm{Si}}, n_{\mathrm{Ge}})$, are determined by calibrating SiGe against its experimental lens-shaped phase diagram (see Figure 2b in the main text). The precise expressions for the energy barriers and the detailed procedure for calibration are reported in Supplementary Note S3.

Solidification at a one-coordinated site can either follow the stacking order dictated by its local atomic environment or it can break it, according to a user-defined probability $P \in [0, 1]$ ($P = 1$ ($P = 0$) means that only cubic (hexagonal) stacking is allowed) [2]. Processes where the interface kinetics is such to destabilize higher-coordinated solid sites, in favor of lower-coordinated ones, are more prone to the formation of stacking defects.

The occupancy, coordination and bonding configuration of any bulk solid site in the super-lattice is constantly accessible in the shared-memory environment over successive FEM-KMCsL cycles. This is crucial to keep track of the amount of solid and liquid species over time, which in turn allows to compute $x_L(t)$ and $x_S(\mathbf{r}, t)$, map the latter into the FEM solver, ensure mass conservation and track the evolution of vacancies (KMCsL voids with $n = 4$ three-coordinated solid neighbours) and stacking disorder throughout the simulation.

FEM model

Continuum modelling in this work consists in using finite element methods (FEM) to solve the heat equation self-consistently with the time-harmonic solution of Maxwell's equations on a mesh, including phase, temperature and alloy-fraction-dependent material parameters [10]. In the context of the multiscale methodology, the mesh is 3D and the solid/liquid phase changes and species redistribution in the liquid and solid phases are modelled by means of KMCsL. In the non-atomistic simulations carried out for consistency and validation analyses, the mesh is 1D and a mixed enthalpy/phase-field formalism is adopted instead [10, 11]. The enthalpy formalism is

used for $T < T_M(x)$, while the phase-field formalism is used for $T > T_M(x)$, with $T_M(x)$ being the melting temperature expected from the phase diagram.

The mesh is always initialized from a TCAD geometry with size of ~20 µm along the direction z of irradiation and 10-30 nm along the lateral x and y (periodic) directions. A 200 nm-thick layer of air is included above the initial surface. The KMCsL-coupled subregion typically includes the top 30-150 nm of the surface and a few-nm layer of air (2 to 10 nm), which needs to be thick enough to accommodate possible resolidification of the material above the initial surface level. The mesh resolution in the KMCsL-coupled subregion is 1-1.5 nm and gradually becomes coarser far from it, until it reaches the mesh lateral size in the top and bottom of the mesh.

The optical and thermal parameters for Si and SiO_2 are taken from Ref. [8], while those for Ge are taken from Refs. [11]. The behavior of SiGe alloy is nearly ideal (Raoultian) [12], hence most of its properties are well approximated by a linear interpolation of pure Si and Ge ones. We recently found that the dielectric function of liquid and solid SiGe requires a more careful definition to properly capture experimentally measured reflectivities [13]. In this work, only for the liquid SiGe dielectric function, we choose for simplicity to use a linear interpolation between pure Si and Ge weighted on x_L, rescaled *ad-hoc* by a factor $a_i \approx 1$ to capture the reflectivity reported in [13]. The a_i and corresponding reflectivity values are reported in Supplementary Table S4.

The models employed for relaxed and strained SiGe only differ in the initial Ge profile and in the definition of solid-phase thermal conductivity. For strained SiGe we refer to the known expression for pure silicon [8]. This is justified by the small thickness of SiGe layers compared to the substrate's in our simulations. For relaxed SiGe we use the expression reported in Ref. [14].

Experiments

Relaxed thick $Si_{1-x}Ge_x$ samples were prepared from two 200 mm bulk Si(0 0 1) wafers (Czochralski, p-type, 1–50 Ω cm). The epitaxy process was performed by Reduced Pressure Chemical Vapor Deposition in a Centura 5200C chamber from Applied Materials. Prior to each SiGe layer epitaxy, a H_2 bake (1373 K, 2 min) was done to remove the native oxide. After the surface cleaning, a graded SiGe buffer layer was grown on each wafer with a 10% / µm ramp (1173 K for one wafer and 1123 K for the other, P = 20 Torr, precursors: SiH_2Cl_2 + GeH_4). Then, 1.2 µm thick relaxed and undoped SiGe layers were grown with a uniform Ge content, corresponding to that of the buffer layer underneath. Thanks to the high temperature used during the process, the glide of the threading arms of misfit dislocations (*i.e.* threading dislocations) was enhanced in such way that

they remained mostly confined in the graded buffer layers, close to the SiGe/Si interface. As a result, the threading dislocations density was significantly reduced in the SiGe top layers ($\sim 10^5$ cm^2). Following the RPCVD process, the remaining cross-hatch patterns were removed using a two steps (planarization and smoothing) Chemical-Mechanical Polishing process thanks to a Mirra CMP system from Applied Materials, reducing the thickness of the SiGe top layers from 1.2 µm to ($\sim$0.7 µm). Nanosecond Laser Annealing was performed with a SCREEN-LASSE (LT-3100) UV laser (λ = 308 nm, single pulse, pulse duration = 160 ns, 4 Hz repetition rate, < 3 % laser beam uniformity, 10×10 mm^2 laser beam) at room temperature and atmospheric pressure, with an constant incident N_2 flux to strongly limit the oxygen incorporation. The Ge composition of laser irradiated SiGe layers was measured with well-calibrated [13] Energy Dispersive X-ray spectroscopy (EDX) in a Transmission Electron Microscope JEM-ARM200F Cold FEG equipped with a EDX SDD CENTURIO-X detector from JEOL. X-ray signals in selected areas have been quantified via the Cliff and Lorimer factor method to extract Ge content profiles as function of depth. The cross-section lamellas were fabricated by Focused Ion Beam in a Helios 450S Scanning Tunneling Electron Microscope from FEI.

NOTE S2. TECHNICAL IMPLEMENTATION OF THE MULTISCALE METHOD

Here we report on the main software engineering tasks needed to enable nanosecond pulsed laser annealing (LA) simulations for SiGe alloys. The means and importance of sharing of RAM between Python and Fortran modules will be deployed. This gives the possibility to ensure mass conservation during the LA process, enable solidification of the material above its as-grown surface level, model extended defects during the process and track the kinetics and redistribution of the various species composing the alloy. The major technical improvement of enabling shared RAM, and thus efficient in-memory data transfers, between the Fortran kinetic monte carlo on super-lattice and the Python continuum FEM environments for LA simulations was achieved by integrating the so-called F2Py sockets [5] into the MulSKIPS code, exploiting some of the routines available in [6]. The workflow after full integration of F2Py functionalities in the Python and Fortran modules is outlined in Figure S1. During a hybrid FEM-KMCsL LA simulation three key data transfers are carried out between the Fortran and Python solvers, one at the beginning of the simulation and the others at every time step Δt of the FEM-KMCsL cycle. The first one is a geometry transfer from Python to Fortran, occurring after interpolation of the relevant part of mesh into the MulSKIPS superlattice formalism. The second one is the thermal field transfer

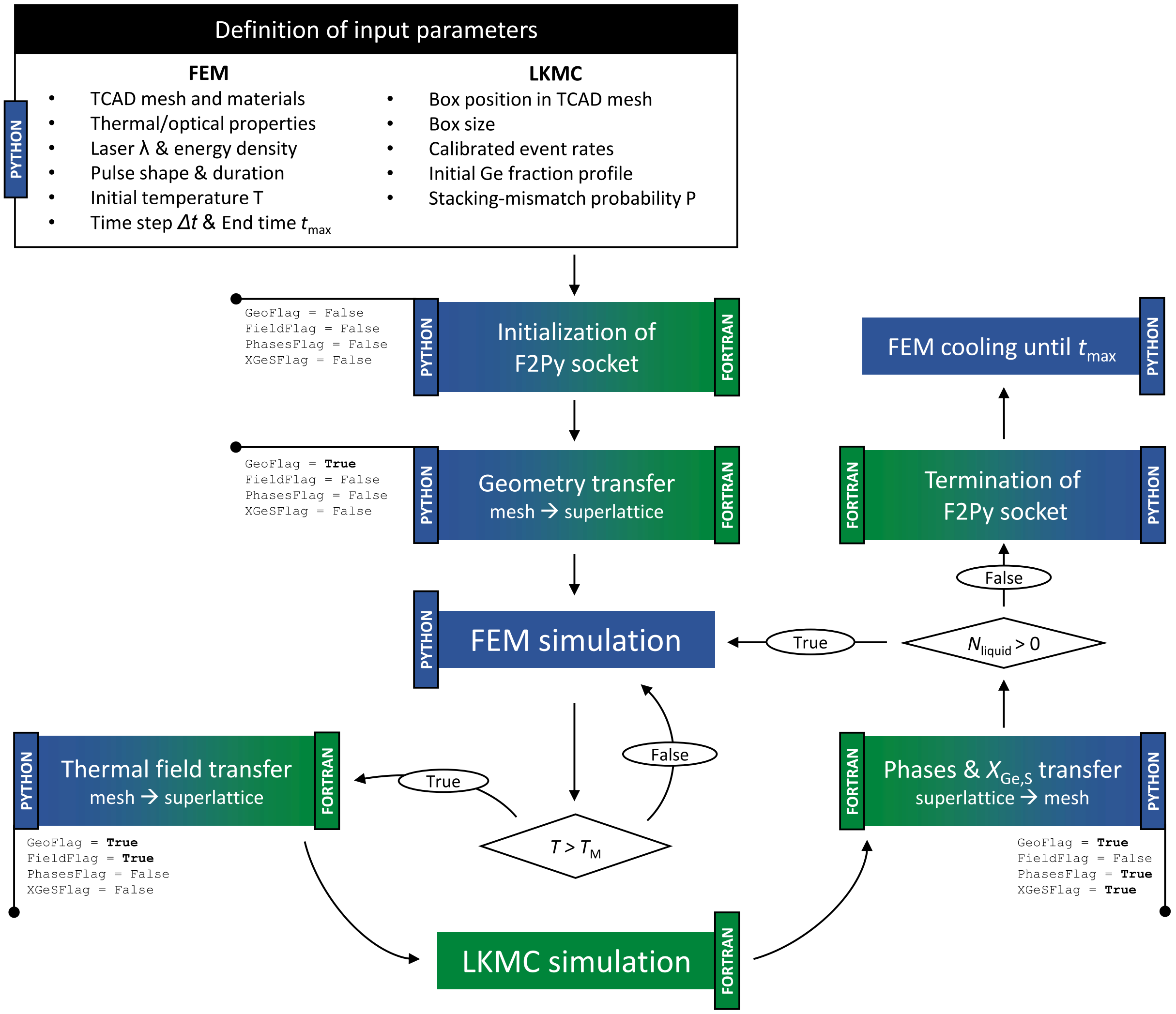

FIG. S1. Technical flowchart of the hybrid atomistic-continuum tool. The required input parameters for FEM and KMCsL models, listed on top, are all provided to a general Python control script. Green and blue colours indicate Fortran and Python-based character of the invoked routines, respectively. Steps directly involving F2Py routines are shown with a colour gradient ranging from green to blue, and vice-versa. The control flags GeoFlag, FieldFlag, PhasesFlag and XGeSFlag are used in Fortran to signal the availability in RAM of the relative data arrays and thus trigger the communications between Fortran and Python.

from Python to Fortran at every time step Δt, which is used as input in MulSKIPS to determine the KMCsL space- and time-dependent melting/solidification event probabilities. The third one is the solid/liquid phases transfer from Fortran to Python at every time step Δt, which is used to get the phase-changed volume in the FEM model and compute the exchanged latent heat needed to solve the Maxwell-Fourier self-consistent problem, and get an updated thermal field in the following iteration. Without implementing F2Py sockets [7], such data (in the form of arrays with KMCsL

superlattice dimension $L_x \times L_y \times L_z$) could only be transferred from Python to Fortran and vice versa by means of hundreds of heavy write/read operations on disk, with obvious performance limitations and demanding storage requirements. F2Py sockets allow performing such array transfers directly in RAM.

From a more technical point of view, the operations involving F2Py sockets are performed by ad-hoc "send" and "receive" routines implemented in both Fortran and Python environments, whose execution is triggered in Fortran by proper combinations of the four control flags GeoFlag, FieldFlag, PhasesFlag and XGeSFlag, each indicating the availability in RAM of the data arrays describing occupancies, thermal field, phases and Ge local fraction, respectively. The initialization of the F2Py socket and the MulSKIPS unique Fortran CPU process is carried out right after setting up the LA simulation parameters in Python. At the beginning of the Fortran CPU process, all flags are set to False, meaning that the KMCsL code is on standby and waiting for instructions from Python. The Python module interpolates a local mesh region into a data array in the form of superlattice sites occupations, issues a "send" command to communicate the geometry information via the F2Py socket, and is put on standby. The "send" command in Python triggers a "receive" command in Fortran, which reads the data array stored in RAM by Python, uses them to set up the superlattice occupations, then sets the GeoFlag to True and is put in standby. In the latter step, the original number of each atomic species is also stored, as a reference to ensure mass conservation during solidification at the latest stages of the LA simulation. The Python module enters the FEM cycle and heats up the system by simulating laser absorption until the maximum temperature in the mesh reaches the trigger temperature melting temperature T_M. At this point, the thermal field is interpolated and sent to Fortran via F2Py sockets, which receive it and set the FieldFlag to True. This last statement, combined with the True state of GeoFlag, triggers the setup of KMCsL probabilities and marks the beginning of the first KMCsL cycle. Once the KMCsL simulated time reaches Δt, the solid/liquid state of each superlattice site and the Si/Ge identities of all solid ones are stored in RAM, a "send" command is issued (this time in Fortran), the PhasesFlag and XGeSflag are set to True and the FieldFlag is restored to False, ensuring that the Fortran CPU process stays in standby until a new thermal field is transferred. Python reads such data from RAM, uses it to update the S/L volumes in the mesh and the solid-phase local Ge fraction, then recalculates the thermal field. As this sequence goes on, the number of liquid and solid sites in the KMCsL superlattice is continuously tracked in the Fortran environment, differentiating the various chemical species (e.g., Si and Ge in SiGe alloys). Once the original number of solid species is recovered (or, equivalently, there are no more liquid sites) the backward

communications to Python occur one last time and the F2Py connection is closed. At this point, the FEM cooling cycle continues until the maximum simulation time $t_{\max}$, set up at the beginning of the LA simulation, is reached.

In addition to optimizing data exchange, an F2Py socket allows for the execution of a unique KMCsL CPU process for the whole LA simulation (rather than a sequence of independent KMCsL calculations reinitialized at every time step Δt). This in turn means that the information about KMCsL sites' occupations and coordinations can be retained across subsequent KMCsL-FEM communication cycles. In Ref. [7] this was unfeasible because MulSKIPS needed to be reinitialized after every FEM step, causing an inevitable reset of MulSKIPS superlattice information.

Overall, RAM storage through F2Py sockets has the important advantages of unlocking the simulation of defects in the irradiated material, which is a native characteristic feature of MulSKIPS . It indeed allows preserving the information about vacancies positions in the KMCsL box across subsequent KMCsL cycles, which would otherwise be lost if MulSKIPS is reinitialized from scratch at every Δt. It makes it possible to keep the information about stacking choices made for all one-coordinated solid sites across subsequent KMCsL Δt-long cycles, enabling the evolution of extended stacking defects during the LA-induced resolidification. Furthermore, it allows tracking the concentration and position of every solid species within the KMCsL box during the LA simulation, which is crucial to count solid sites at every instant of the simulation and ensure that solidification ends whenever the original solid mass is recovered.

NOTE S3. DETAILS ON KMCSL CALIBRATION

TABLE S1. Solidification energies for Si (left column) and Ge (right column). The energy values E_2^{Si} and E_2^{Ge} are obtained by calibrating the pure species.

Si	Ge
$E_{\mathrm{LS}}^{\mathrm{Si}}(1) = (E_2^{\mathrm{Si}} - \delta^{\mathrm{Si}})$	$E_{\mathrm{LS}}^{\mathrm{Ge}}(1) = (E_2^{\mathrm{Ge}} - \delta^{\mathrm{Ge}})$
$E_{\mathrm{LS}}^{\mathrm{Si}}(2) = E_2^{\mathrm{Si}}$	$E_{\mathrm{LS}}^{\mathrm{Ge}}(2) = E_2^{\mathrm{Ge}}$
$E_{\mathrm{LS}}^{\mathrm{Si}}(3) = (E_2^{\mathrm{Si}} + \delta^{\mathrm{Si}})$	$E_{\mathrm{LS}}^{\mathrm{Ge}}(3) = (E_2^{\mathrm{Ge}} + \delta^{\mathrm{Ge}})$

TABLE S2. Melting energies $E_{\mathrm{SL}}^{i}(n_{\mathrm{Si}}, n_{\mathrm{Ge}})$ for i=Si (left column) and i=Ge (right column).

Si	Ge
$E_{\mathrm{SL}}^{\mathrm{Si}}(1,0) = E_2^{\mathrm{Si}}$	$E_{\mathrm{SL}}^{\mathrm{Ge}}(1,0) = (1+\beta)[E_2^{\mathrm{Ge}} + E_2^{\mathrm{Si}}]/2$
$E_{\mathrm{SL}}^{\mathrm{Si}}(0,1) = (1-\alpha)[E_2^{\mathrm{Si}} + E_2^{\mathrm{Ge}}]/2$	$E_{\mathrm{SL}}^{\mathrm{Ge}}(0,1) = E_2^{\mathrm{Ge}}$
$E_{\mathrm{SL}}^{\mathrm{Si}}(1,1) = (1-\alpha)[3E_2^{\mathrm{Si}} + E_2^{\mathrm{Ge}}]/4$	$E_{\mathrm{SL}}^{\mathrm{Ge}}(1,1) = (1+\beta)[3E_2^{\mathrm{Ge}} + E_2^{\mathrm{Si}}]/4$
$E_{\mathrm{SL}}^{\mathrm{Si}}(2,0) = E_2^{\mathrm{Si}}$	$E_{\mathrm{SL}}^{\mathrm{Ge}}(2,0) = (1+\beta)[E_2^{\mathrm{Ge}} + E_2^{\mathrm{Si}}]/2$
$E_{\mathrm{SL}}^{\mathrm{Si}}(0,2) = (1-\alpha)[E_2^{\mathrm{Si}} + E_2^{\mathrm{Ge}}]/2$	$E_{\mathrm{SL}}^{\mathrm{Ge}}(0,2) = E_2^{\mathrm{Ge}}$
$E_{\mathrm{SL}}^{\mathrm{Si}}(2,1) = (1-\alpha)[5E_2^{\mathrm{Si}} + E_2^{\mathrm{Ge}}]/6$	$E_{\mathrm{SL}}^{\mathrm{Ge}}(2,1) = (1+\beta)[2E_2^{\mathrm{Ge}} + E_2^{\mathrm{Si}}]/3$
$E_{\mathrm{SL}}^{\mathrm{Si}}(1,2) = (1-\alpha)[2E_2^{\mathrm{Si}} + E_2^{\mathrm{Ge}}]/3$	$E_{\mathrm{SL}}^{\mathrm{Ge}}(1,2) = (1+\beta)[5E_2^{\mathrm{Ge}} + E_2^{\mathrm{Si}}]/6$
$E_{\mathrm{SL}}^{\mathrm{Si}}(3,0) = E_2^{\mathrm{Si}}$	$E_{\mathrm{SL}}^{\mathrm{Ge}}(3,0) = (1+\beta)[E_2^{\mathrm{Ge}} + E_2^{\mathrm{Si}}]/2$
$E_{\mathrm{SL}}^{\mathrm{Si}}(0,3) = (1-\alpha)[E_2^{\mathrm{Si}} + E_2^{\mathrm{Ge}}]/2$	$E_{\mathrm{SL}}^{\mathrm{Ge}}(0,3) = E_2^{\mathrm{Ge}}$

TABLE S3. KMCsL calibrated parameters for pure Ge and Si.

-	Ge	Si
n_0	$2.60 \cdot 10^{16}$	$1.33 \cdot 10^{17}$
E_2 [eV]	0.65	0.96
δ [eV]	0.02	0.03
T_0 [K]	900	1080
A [K]	210	280
T_M [K]	1210	1688

The expressions of the energy barriers appearing in Equation (S1) and Equation (S2) are reported in Table S1 and Table S2 for solidification and melting events, respectively.

The calibration of Si-Ge mixtures is based on the single-species calibrations of pure Si and Ge. All the parameters involved in Equation (S1) and Equation (S2), except for the melting energy

barriers involving mixed bonds (i.e., $E^{\rm Si}_{\rm SL}(n_{\rm Si}, n_{\rm Ge} \neq 0)$ and $E^{\rm Ge}_{\rm SL}(n_{\rm Si} \neq 0, n_{\rm Ge})$), were calibrated against the Fulcher-Vogel relation for the pure (crystalline) species, which gives the temperature dependence of the interface velocity [8, 9]. The results are reported in Table S3.

For SiGe, since a Fulcher-Vogel relation cannot be defined for alloys, we relied on the experimental phase diagram of the material, expressing the temperature-dependent composition of the solid and the liquid phases at equilibrium, when the melting/solidification process is operated at a very slow speed [12]. The behavior of the alloy is very close to ideal (Raoultian), where enthalpic contributions to the mixing energy are negligible and the lens shape of the diagram mostly depends on the entropy of fusion of the component species (the larger the entropy, the broader the shape). Under such assumption of ideal mixing, the energy E_{ij} of a mixed bond can be estimated as $E_{ij} = ((E_{ii} + E_{jj}))/2$. An initial guess for the values of $E^{i}_{\rm SL}(n_{\rm Si}, n_{\rm Ge})$ is therefore obtained as linear combinations of the solidification energy barriers for pure Si and Ge, $E^{\rm Si}_2 \equiv E^{\rm Si}_{\rm LS}(2)$ and $E^{\rm Ge}_2 \equiv E^{\rm Ge}_{\rm LS}(2)$ weighed on the number of Si and Ge nearest neighbors. The energy barriers for mixed bonding states are perturbed (i.e., further decreased in the case of Si and increased in the case of Ge) by a small amount to reproduce the experimental phase diagram of the alloy. The resulting 18 energy parameters (9 for Si and 9 for Ge) take the form reported in Table S2. For the sake of simplicity, the perturbation terms α and β were chosen to be the same for every coordination state of Si and Ge, respectively, thus reducing the calibration to such two parameters only. For calibration purposes, a few assumptions were made in the simulation set-up. The liquid phase surmounting the solid material is assumed to have a fixed Ge fraction x_L, meaning negligible diffusion times of Si and Ge atoms within an infinite liquid reservoir. The temperature is kept constant and uniform in the simulation box at every KMCsL run. We note that in the FEM-KMCsL LA simulations the KMCsL event rates in Equation (S1) and Equation (S2) are functions of time t and lattice position $\mathbf{r}$. This is because the condition of fixed and uniform temperature in the simulation box is dropped and the liquid-phase composition $x_L \equiv x_L(t)$ (i.e., the parameter X^i) becomes a time-dependent variable. The overall workflow of the calibration is described below:

- Set the calibration parameters α and β.
- Set the desired molar fraction of Ge in the liquid phase, x_L, which is assumed to be fixed during the KMCsL run and set to be equal to the initial solid seed (the latter assumption is arbitrary, since the composition of the solidified bulk material will depend on x_L and on the KMCsL event probabilities).
- Run MulSKIPS in the temperature interval $[T^{\rm exp}_M - 100, T^{\rm exp}_M + 100]$, where $T^{\rm exp}_M$ is the

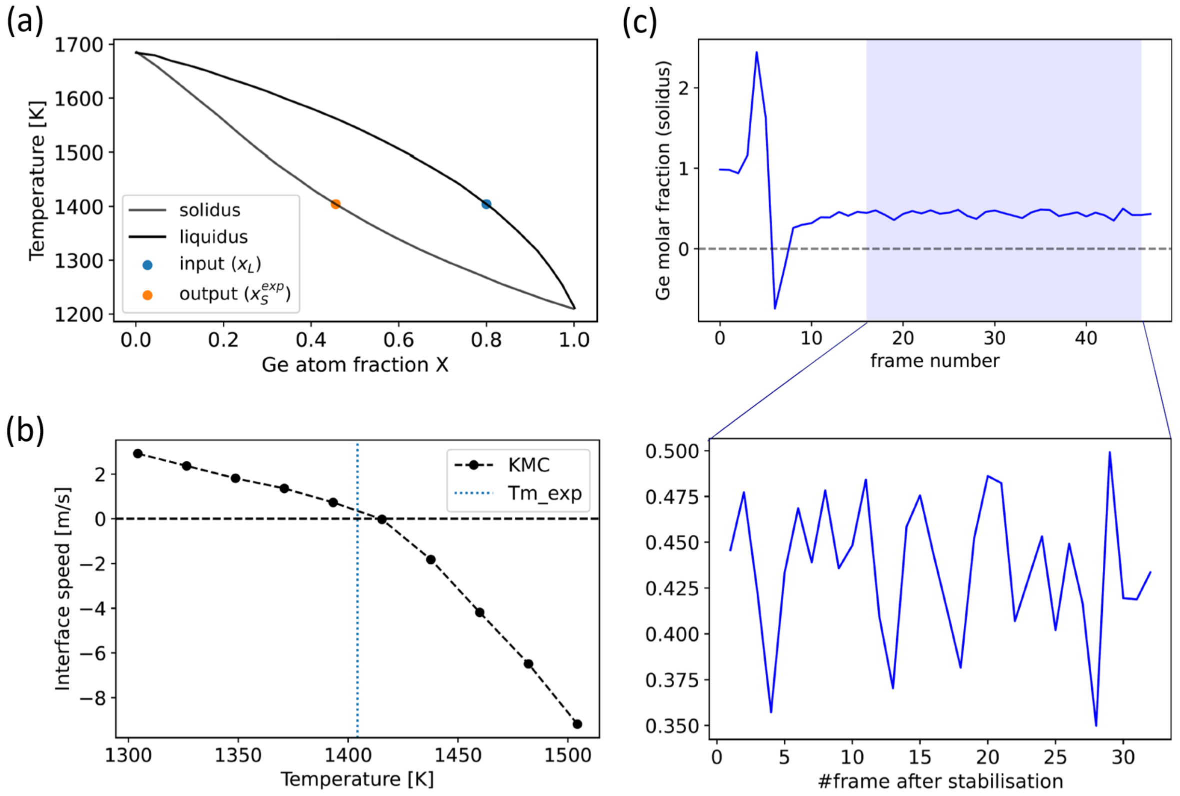

FIG. S2. Some steps of the KMCsL calibration workflow for the case of $x_L = 0.8$. (a) experimental phase diagram of the SiGe mixture. The x_L value on the liquidus curve is marked in blue, the expected $x_S^{\rm exp}$ value on the solidus curve (0.46 in this case) is marked in orange. (b) temperature-dependent interface speed predicted from the KMCsL model (black dashed line). The expected equilibrium temperature $T_M^{\rm exp}$ at x_L extracted from the phase diagram is shown as blue dotted line. The equilibrium temperature $T_M^{\rm KMCsL}$ interpolated from the KMCsL curve is 1415 K. (c) Evolution of the bulk Ge fraction obtained from the KMCsL run at $T_M^{\rm run}$ =1410K; the initial frames are discarded until the $x_S^{\rm KMCsL}$ value stabilises around a constant value. In this case, $x_S^{\rm KMCsL} = 0.44$, with standard deviation $\sigma(x_S^{\rm KMCsL}) = 0.04$.

equilibrium temperature in phase diagram, corresponding to $x = x_L$ (see Figure S2a). The equilibrium temperature, $T_M^{\rm KMCsL}$, is interpolated as the temperature corresponding to zero interface velocity in the KMCsL runs (see Figure S2b).

- Run MulSKIPS at a constant temperature $T^{\rm run}$ slightly lower than $T_M^{\rm KMCsL}$ (by 5 K), to ensure quasi-equilibrium solidification of the material.

- The Ge molar fraction of the solidified material, $x_S^{\rm KMCsL} = n_{\rm Ge}/(n_{\rm Si}+n_{\rm Ge})$, and the standard deviation $\sigma(x_S^{\rm KMCsL})$, are calculated by tracking the number of Si and Ge atoms solidified at every time frame in the KMCsL cycle, and averaging the Ge fraction once the solidification

is stabilized (see Figure S2c).

- Compare the KMCsL results to the experimental phase diagram. Repeat the steps above with updated α and β, if necessary.

The calibration yielded α=0.06 and β=0.08, suggesting a small (less than 10%) perturbation to the Raoultian behavior of the mixture.

NOTE S4. DIFFERENCES BETWEEN PHASE-FIELD AND FEM-KMCSL APPROACHES

Besides the trivial difference in dimensionality and in the modelling approach to phase transitions, other technical differences exist between the phase-field and the multiscale FEM-KMCsL methodology, which are at the origin of the deviations observed in Figure 3 of the main text.

1. Melting in phase-field simulations occurs at $T \geq T_M(x_L)$, with $T_M(x_L)$ being the liquidus line of the phase diagram drawn in Figure 2 of the main text (dashed lines). In the FEM-KMCsL simulations, local melting is governed by the balance between the well-calibrated KMCsL melting and solidification events.

2. The interface smoothness within the phase-field formalism is user-defined. The melt front evolution in planar samples is tracked by looking at the flex of the phase function as a function of depth. Both this phase profile and the local Ge concentration profile in the mesh are seamless and smooth step-like functions of depth, uniquely defined across solid and liquid regions. In the KMCsL formalism the solid/liquid (S/L) interface is atomically sharp, with its position in planar samples evaluated as the average z coordinate of all undercoordinated solid atoms. This different smoothness is at the origin of the slightly higher (6 nm) maximum melt depth found with FEM-KMCsL for 30nm strained SiGe irradiated with high energy density (i.e., 1.9 J cm^{-2}). Here there is an instant where the S/L interface falls below the initial SiGe/Si junction. At this point, in the phase-field simulation, the Ge concentration drops to zero in a gradual fashion. Instead, in the FEM-KMCsL simulation, a higher concentration of Ge (i.e., lower T_M) exists in the liquid region right above the SLI (due to its much more step-like Ge profile). The heat to be released to stop melting and initiate solidification is therefore higher in the FEM-KMCsL than in phase-field simulations.

3. Atomic sites in the liquid phase are not explicitly implemented in the current KMCsL framework, and neither are liquid-phase diffusion events. Si and Ge concentrations are computed as averages, by direct subtraction of the number of Si and Ge solid sites from the respective initial amounts. On one hand, this averaging is justified by the high value of Ge diffusivity in liquid SiGe near melting temperature (10^{-4} cm^2/s [8, 15]), which yields Ge diffusion velocities 3-4 times higher than the typical solidification velocity in LA processes with ns pulses (2-3 m/s) [16, 17]. In fact, melt depths and overall Ge segregation trends are well reproduced. On the other hand, approximating as infinite such a high diffusivity leads to

underestimated Ge trapping at the beginning of solidification, compared to phase-field. This is evident from the different minima found in the post-anneal Ge profiles of Figure 3g-h of the main text. In turn, FEM-KMCsL simulations always end with the solidification of a pure Ge capping layer (x=1) with an energy density-dependent thickness, whereas phase-field simulations provide a SiGe capping layer with energy density-dependent Ge content and an almost constant thickness. The variation of capping layer thickness in FEM-KMCsL simulations explains the the energy density-dependent plateaus found in the KMCsL melt depth profiles. The variation in Ge content in the phase-field simulations explains the energy density-dependent plateaus found in the phase-field $T_{\max}$ profiles.

4. The purely continuum methodology presents numerical instabilities at the onset of melting and at the end of solidification. For example, one may notice the abrupt melt depth variation in the latest stage of solidification in Figure 3c of the main text. These instabilities are due to the switching between enthalpy and phase-field continuum solvers occurring every time T crosses $T_M(x_L)$. A good convergence of the simulations needs to be achieved, by proper adjustments of the time step Δt, interface smoothness and threshold size for nucleation. The only numerical instabilities affecting the FEM-KMCsL simulations concern temperature oscillations, like the small ones observed at the onset of melting in Figure 3e of the main text. These are due to the small (1.5 nm) but finite thickness of the liquid layer initially defined at nucleation stage to stably initialize the melting phenomenon. Such oscillations can be reduced by setting a smaller Δt [7], as confirmed by the results for strained SiGe, where $\Delta t = 0.25$ ns was used, instead of 0.5 ns.

NOTE S5. CORRECTION TO THE LIQUID SIGE DIELECTRIC CONSTANT

TABLE S4. Correction factors a_i used in the linearly interpolated expression for the dielectric constant of liquid SiGe, $[\epsilon_{l,SiGe}(x_{Ge}) = \epsilon_{l,Ge} \cdot x_{Ge} + \epsilon_{l,Si} \cdot (1 - x_{Ge})] \cdot a_i$, in order to reproduce the experimental melt depths in Figure 4 of main text. The values of reflectivity are also reported, before ($R_{a_i} = 0$) and after (R_{a_i}) the correction. ED stands for energy density.

Sample	Δt_{pulse} [ns]	ED [J cm^{-2}]	a_i	R_{a_i}	$R_{a_i=0}$
Relaxed $Si_{0.76}Ge_{0.24}$	160	0.75	1.5627	0.815	0.778
	160	1.10	1.4160	0.807	0.778
Relaxed $Si_{0.42}Ge_{0.58}$	160	0.90	1.3258	0.802	0.775
Strained $Si_{0.8}Ge_{0.2}$	146	1.80	1.1938	0.794	0.778
	146	2.00	1.4073	0.807	0.778
Strained $Si_{0.6}Ge_{0.4}$	146	1.60	1.4073	0.769	0.768
	146	1.81	1.2460	0.797	0.768

NOTE S6. LA SIMULATION RESULTS FOR ADVANCED SIGE SYSTEMS

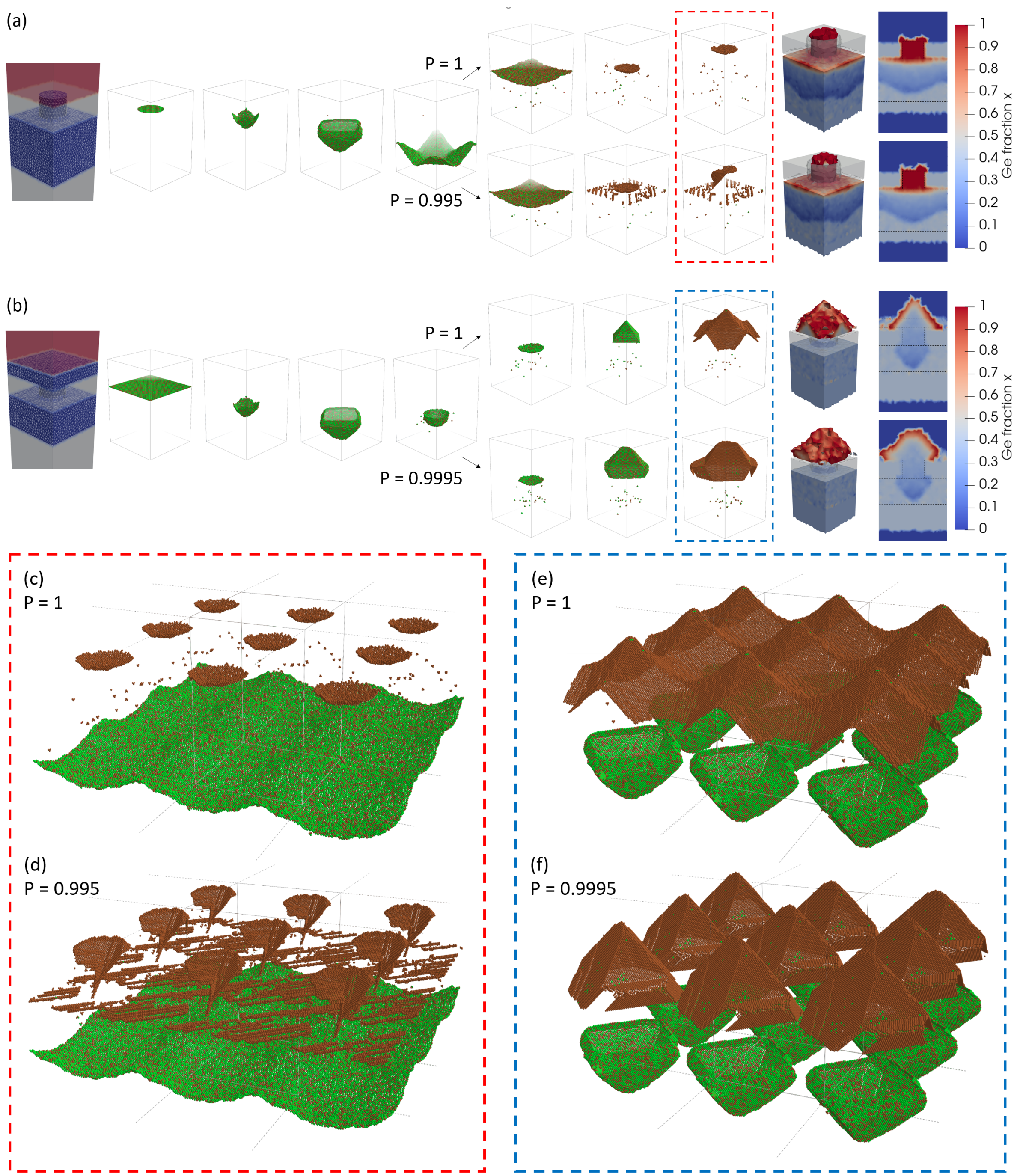

FIG. S3. (a-b) Same simulations of Figure 6 in main text, but with KMCsL snapshots not overlapped. A 3 × 3 periodic repetition of the KMCsL interface at the end of melting and solidification stages in the simulations in (a) is shown in (c) for P=1 and in (d) for P=0.995. For the simulations in (b), the KMCsL cell is reported in (e) for P=1 and in (f) for P=0.9995.

REFERENCES

[1] G. Calogero, I. Deretzis, G. Fisicaro, and A. La Magna, "Mulskips," `https://github.com/MulSKIPS`, accessed 2023-09-13.

[2] A. La Magna, A. Alberti, E. Barbagiovanni, C. Bongiorno, M. Cascio, I. Deretzis, F. La Via, and E. Smecca, Phys. Status Solidi A **216**, 1800597 (2019).

[3] G. Fisicaro, C. Bongiorno, I. Deretzis, F. Giannazzo, F. La Via, F. Roccaforte, M. Zielinski, M. Zimbone, and A. La Magna, Appl. Phys. Rev. **7**, 021402 (2020).

[4] M. Alnæs, J. Blechta, J. Hake, A. Johansson, B. Kehlet, A. Logg, C. Richardson, J. Ring, M. E. Rognes, and G. N. Wells, Archive of Numerical Software **3**, 100 (2015).

[5] P. Peterson, International Journal of Computational Science and Engineering **4**, 296 (2009).

[6] B. F. Garcia, "f2py-sockets," `https://github.com/b-fg/f2py-sockets`, accessed 2023-09-13.

[7] G. Calogero, D. Raciti, P. Acosta-Alba, F. Cristiano, I. Deretzis, G. Fisicaro, K. Huet, S. Kerdilès, A. Sciuto, and A. L. Magna, npj Computational Materials **8** (2022), 10.1038/s41524-022-00720-y.

[8] A. La Magna, P. Alippi, V. Privitera, G. Fortunato, M. Camalleri, and B. Svensson, J. Appl. Phys. **95**, 4806 (2004).

[9] A. Mittiga, L. Fornarini, and R. Carluccio, Applied Surface Science **154-155**, 112 (2000).

[10] F. Cristiano and A. La Magna, eds., *Laser Annealing Processes in Semiconductor Technology* (Woodhead Publishing, 2021).

[11] K. Huet, J. Aubin, P.-E. Raynal, B. Curvers, A. Verstraete, B. Lespinasse, F. Mazzamuto, A. Sciuto, S. Lombardo, A. La Magna, P. Acosta-Alba, L. Dagault, C. Licitra, J.-M. Hartmann, and S. Kerdilès, Appl. Surf. Sci. **505**, 144470 (2020).

[12] R. W. Olesinski and G. J. Abbaschian, Bulletin of Alloy Phase Diagrams **5**, 180 (1984).

[13] D. Ricciarelli, G. Mannino, I. Deretzis, G. Calogero, G. Fisicaro, R. Daubriac, F. Cristiano, R. Demoulin, P. P. Michałowski, P. Acosta-Alba et al., Materials Science in Semiconductor Processing **165**, 107635 (2023).

[14] M. Wagner, G. Span, S. Holzer, O. Triebl, T. Grasser, and V. Palankovski, ECS Transactions **3**, 1151 (2006).

[15] Y. Cai, G. Zhang, and H. Fu, Journal of Applied Physics **87**, 7735 (2000).

[16] L. Dagault, *Investigation of Si1-XGex epilayers behavior upon ultraviolet nanosecond laser annealing*, Ph.D. thesis, Université Toulouse 3 Paul Sabatier (2021).

[17] P. Baeri and E. Rimini, Materials Chemistry and Physics **46**, 169 (1996).
